# Metabolomic Profiling of Angiotensin-II-Induced Abdominal Aortic Aneurysm in Ldlr^−/−^ Mice Points to Alteration of Nitric Oxide, Lipid, and Energy Metabolisms

**DOI:** 10.3390/ijms23126387

**Published:** 2022-06-07

**Authors:** Juan Manuel Chao de la Barca, Alexis Richard, Pauline Robert, Maroua Eid, Olivier Fouquet, Lydie Tessier, Céline Wetterwald, Justine Faure, Celine Fassot, Daniel Henrion, Pascal Reynier, Laurent Loufrani

**Affiliations:** 1 UMR CNRS 6015—INSERM U1083, IRIS2, 3 rue Roger Amsler, 49100 Angers, France; jmchaodelabarca@chu-angers.fr (J.M.C.d.l.B.); alexis.richard@univ-angers.fr (A.R.); pauline.robert@univ-angers.fr (P.R.); celine.fassot@inserm.fr (C.F.); daniel.henrion@univ-angers.fr (D.H.); pareynier@chu-angers.fr (P.R.); 2 INSERM U1083, 49100 Angers, France; 3 Mitovasc Institute, Université d’Angers, 49100 Angers, France; 4 Service de Biochimie et Biologie Moléculaire, Centre Hospitalier Universitaire (CHU), 49000 Angers, France; l.tessier@chu-angers.fr (L.T.); c.wetterwald@chu-angers.fr (C.W.); j.faure@chu-angers.fr (J.F.); 5 Service de Chirurgie Cardiaque, Centre Hospitalier Universitaire (CHU), 49100 Angers, France; maroua.eid@chu-angers.fr (M.E.); olfouquet@chu-angers.fr (O.F.); 6 Angers University Hospital (CHU), 49100 Angers, France

**Keywords:** aneurysm, blood flow, hypertension, aorta, metabolomics, lipidomics

## Abstract

Aneurysm is the second-most common disease affecting the aorta worldwide after atherosclerosis. While several clinical metabolomic studies have been reported, no study has reported deep metabolomic phenotyping in experimental animal models of aortic aneurysm. We performed a targeted metabolomics study on the blood and aortas of an experimental mice model of aortic aneurysm generated by high-cholesterol diet and angiotensin II in Ldlr^−/−^ mice. The mice model showed a significant increase in media/lumen ratio and wall area, which is associated with lipid deposition within the adventitia, describing a hypertrophic remodeling with an aneurysm profile of the abdominal aorta. Altered aortas showed increased collagen remodeling, disruption of lipid metabolism, decreased glucose, nitric oxide and lysine metabolisms, and increased polyamines and asymmetric dimethylarginine (ADMA) production. In blood, a major hyperlipidemia was observed with decreased concentrations of glutamine, glycine, taurine, and carnitine, and increased concentrations of the branched amino acids (BCAA). The BCAA/glycine and BCAA/glutamine ratios discriminated with very good sensitivity and specificity between aneurysmatic and non-aneurysmatic mice. To conclude, our results reveal that experimental induction of aortic aneurysms causes a profound alteration in the metabolic profile in aortas and blood, mainly centered on an alteration of NO, lipid, and energetic metabolisms.

## 1. Introduction

Aneurysm is the second-most common world disease affecting the aorta after atherosclerosis. It is also the 15th leading cause of death in people over 55 and the 19th leading cause of death worldwide [1]. Aneurysms are defined anatomically as localized arterial wall dilation and functionally as a progressive loss of the capacity of the arterial wall to support its hemodynamic load, which can lead to wall rupture. As aneurysms are usually asymptomatic, detection often occurs incidentally, mainly following clinical imaging.

Today, abdominal aortic aneurysm (AAA), defined by an aortic diameter ≥3 cm, has a high rate of incidence (75% of aortic aneurysms), with a mortality rate over 80%, due to parietal rupture [2]. In addition to this high mortality risk, patients with AAA have deleterious comorbidities/risk factors such as arterial hypertension, dyslipidemia, or metabolic syndrome. Furthermore, due to being associated with arterial hypertension, AAA is accentuated by plasma solute convection involved in extracellular matrix degradation associated with the MMP (matrix metalloproteinase)/TIMP (tissue inhibitor of metalloproteinases) protease system, VSMC (vascular smooth muscle cell) apoptosis, oxidative stress, aging processes, and the adventitial immune response. This is exacerbated by an intraluminal thrombus development [3].

According to studies of population-based AAA screening, AAA prevalence is dependent on age, sex, and geographic location [4]. Efforts to limit the mortality rate of ruptured AAA depend on early detection of AAA. No well-defined treatment strategy is proved, and large-scale randomized trials are still required [5,6]. Surgical repair is currently indicated when the AAA size is greater than 5.5 cm in diameter, and it was shown that survival was not increased by surgical repair for small AAA (<5.5 cm in diameter) [6]. Due to the potentially fatal consequences of arterial dissections and ruptures of aneurysms, there is considerable interest in developing early diagnostic tests.

Interest in the mechanistic study of AAA to respond to this urgency is topical. Even in the absence of accurate knowledge of human AAA, animal models of AAA are necessary. There are different models of AAA, such as the model induced by the peri-aortic application of CaCl_2_ [7], by intraluminal infusion [8], or by the peri-aortic application of elastase [9]. These different models are “artificial” models of AAA, emphasizing one of the actors of pathogenesis, which is the enzymatic degradation of the arterial wall. They do not reproduce the pathology of AAA as completely as the mouse model Ldlr^−/−^ [10] fed with a diet rich in cholesterol and subjected to treatment with angiotensin II. Indeed, unlike human aneurysms and the Ldlr^−/−^ model, AAA induced by product application is not associated with the presence of intra-luminal thrombus, atherosclerosis and rarely lead to rupture. According to literature, the use of a Ldlr^−/−^ model is more appropriate to study the various metabolic disturbances because it is closest to the dyslipidemia found in humans with abdominal aortic aneurysm (AAA) [11,12] and increases the incidence of AAA compared to the ApoE^−/−^ model [13]. Whether this Ldlr^−/−^ model was fed with a fat-enriched diet or not and whether it was subjected or not to treatment with angiotensin II [14] are important to obtaining important information from a signaling point of view on the human disease.

A fascinating aspect of AAAs which developed in these mice is their location in the suprarenal segment of the aorta, reflecting an inherent property of the arterial wall that would favor the incidence of aneurysms there. This may be caused by the heterogeneity of intracellular responses in different regions of the aorta, which may be attributable to the different origins of smooth muscle cells [15]. Nevertheless, comprehension of metabolic and biochemical interactions in this pathology is essential to guaranteeing a better understanding of its pathological mechanisms.

Metabolomics-based deep phenotyping is a promising data-driven approach to identifying biomarkers for early diagnosis of aneurysms as well as to predicting their risk of dissection or rupture. Metabolomics is a high-throughput omics technology that succeeds genomics, transcriptomics, and proteomics, allowing the simultaneous measurement of large sets of small molecules (<1000 Da) [16].

Metabolomic studies to date have mainly been performed on the blood or aortas of patients. In surgically recovered aortas, one study compared aortic aneurysms with early atherosclerotic lesions [17]. This study revealed an upregulation of kynurenine metabolism involving tryptophan, kynurenin, and quinolinic acid, suggesting that macrophage kynureninase may negatively regulate inflammation in aortic atherosclerotic aneurysms. Another study comparing bicuspid and tricuspid aortic-valve-associated aneurysms to age-matched controls revealed five discriminant metabolites with a general increase in the amount of sphingomyelins concentrations, suggesting a repression of sphingomyelinase activity [18].

In blood, Lieberg et al. compared slow- to fast-growing abdominal aortic aneurysms to a healthy control population [19]. Four amino acids (histidine, asparagine, leucine, and isoleucine) and four phosphatidylcholines were found to be significantly altered in the aneurysms compared to the control population, whereas no discriminating metabolites were identified between patients with slow- and fast-growing aneurysms. Another study in blood investigated whether the size of abdominal aortic aneurysms impacted the metabolome of patients by comparing patients with large (>5 cm) and small (<5 cm) aneurysms to a population of healthy controls [20]. This study found that sphingolipids, lysophosphatidylcholines, metabolites of cholesterol, and acylcarnitines discriminated in blood between these two groups at different stages of aneurysm development. Lastly, three other blood studies focused on aortic dissection. Ren et al. compared patients with aortic dissection against healthy subjects and patients with hypertension [21]. One hundred forty metabolites were found to be discriminating in blood between aortic dissections and the hypertensive control group, most of which were involved in the metabolism of tryptophan, histidine, glycerophospholipids, and choline. Stanford classification categorizes aortic dissections according to their anatomic location, and Zhou et al. compared the plasma metabolome of controls and patients with acute aortic dissection divided into two subgroups following the Stanford classification [22]. This study revealed that several sphingolipids and ceramides had altered concentrations between patients and controls and that they were dramatically decreased in the subgroup of patients with Stanford type A. Finally, Yang’s study compared the blood metabolome of patients with thoracic aortic dissection to patients with undissected thoracic aortic aneurysms and to healthy controls [23]. This study revealed an involvement of ceramides in aortic dissection and particularly C18-ceramide, suggesting a possible involvement of this ceramide in macrophage inflammation and MMP expression.

Surprisingly, we have not identified any published metabolomic studies to date on animal models of aneurysms. Furthermore, none of the published metabolomic studies in humans have been conducted on both blood and aortic biopsies. Thus, the coordinated study of these two samples in an animal model could be very useful for pathophysiological research and the search for innovative biomarkers. The present study analyzed the metabolomic profiles obtained from the blood and aorta tissue of an experimental model of aortic aneurysm generated by high cholesterol diet and angiotensin II in a *Ldlr*^−/−^ mouse model.

## 2. Results

### 2.1. Hypertension Induces Cardiac Remodeling in Mice Fed a Cholesterol-Enriched Diet

**Figure 1:**
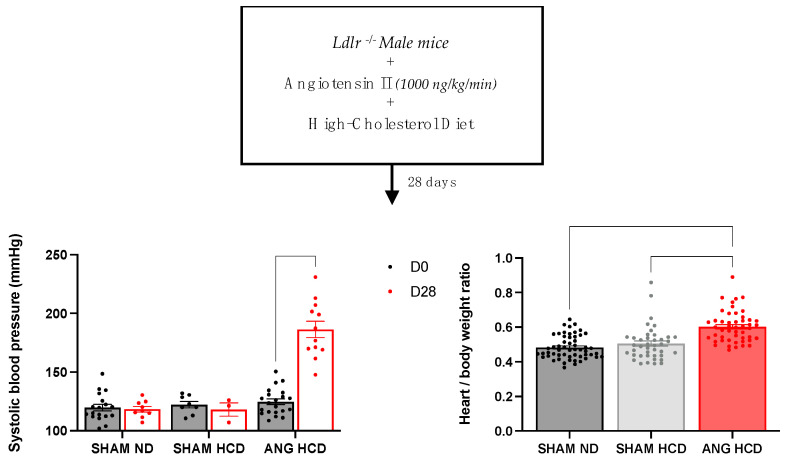
Blood pressure and cardiac remodeling in *Ldlr*^−/−^ mice with or without hypercholesterolemic diet and angiotensin II treatment. Data are given as the mean ± SEM. Two-way ANOVA tests with Bonferroni correction were used for systolic blood pressure (*n* = 8–21), and Kruskal–Wallis tests with Dunn correction for heart/body weight ratio (*n* = 49–55).

Mouse blood pressure was found to be homogeneous between the three groups in a basal state (Figure 1). Hypertensive treatment by angiotensin II induced a significant increase in systolic blood pressure (SBP) in the groups subjected to a high-cholesterol diet (125 ± 2 vs. 186 ± 7 mmHg; *p* < 0.0001). In addition, angiotensin II induced an increase in cardiac mass in mice on the high-cholesterol diet (0.48 ± 0.009 and 0.5 ± 0.02 vs. 0.6 ± 0.01 g; *p* < 0.0001).

### 2.2. Lipid Profile in Ldlr^−/−^ Mice with or without Hypercholesterolemic Diet and Angiotensin II Treatment

As described in Table 1, *Ldlr*^−/−^ mice with cholesterol-enriched diets associated with angiotensin II treatment tend to increase circulating cholesterol and circulating LDL (23.4 ± 1.041 vs. 6.82 ± 1.29 mmol/L; *p* < 0.05) compared to mice fed a standard diet.

Moreover, mice fed a high-cholesterol diet with or without angiotensin II treatment do not present any change in circulating HDL compared to a standard diet. Nevertheless, a significant decrease in plasma triglycerides (4.58 ± 0.83 vs. 0.99 ± 0.1; *p* < 0.05) is established.

### 2.3. Hypertrophic Remodelling with Lipid Deposition of Abdominal Aorta in Ldlr^−/−^ Mice with High-Cholesterol Diet and Angiotensin II Treatment

**Figure 2:**
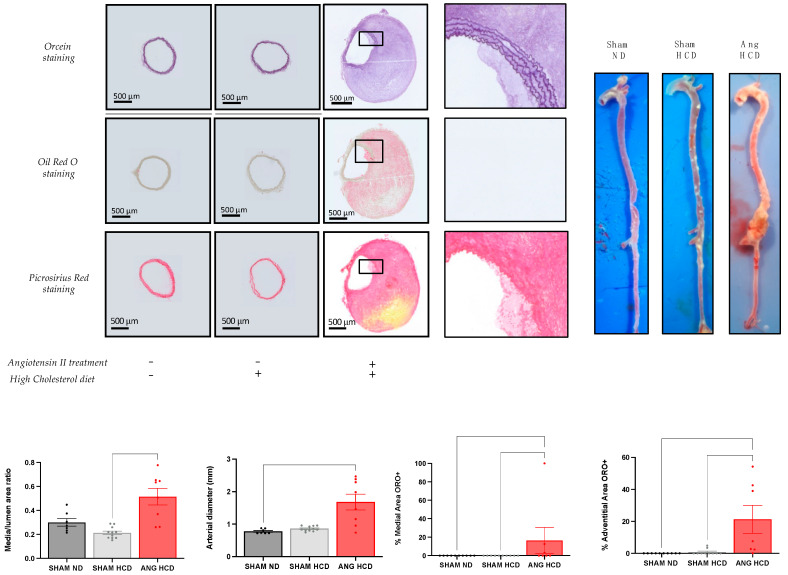
Histomorphologic analysis of abdominal aorta in *Ldlr*^−/−^ mice with or without hypercholesterolemic diet and angiotensin II treatment. ORO+: positive Oil Red O staining. Data are given as the mean ± SEM. Kruskal–Wallis tests with Dunn correction were used.

Mice with angiotensin II treatment and fed a high-cholesterol diet showed a significant increase in media/lumen ratio (0.21 ± 0.015 vs. 0.51 ± 0.07; *p* < 0.001) compared to SHAM mice with high cholesterol diet (Figure 2). In addition, they presented a significant increase in aortic diameter (0.7767 ± 0.02706 vs. 1.678 ± 0.2434 mm; *p* < 0.01) associated with lipid deposition within the adventitia (0 vs. 21.23 ± 8.727%; *p* < 0.001) and media (0 vs. 16.54 ± 14.02%; *p* < 0.05) compared to standard-diet-fed mice, describing a hypertrophic remodeling with an atheromatous profile of the abdominal aorta.

### 2.4. Metabolomic Data Analysis

#### 2.4.1. Analysis of Plasma and Aorta Metabolomes

After validation of the three quality controls (QCs), 154 and 136 metabolites were correctly measured in plasma and aorta samples, respectively, and kept for the statistical analysis (see Supplementary Data S1 and S2 for plasma and aortas, respectively). Plasma and aorta data blocks were submitted to multiblock orthogonal component analysis (MOCA) after normalizing concentrations of aorta samples by their weights.

**Figure 3:**
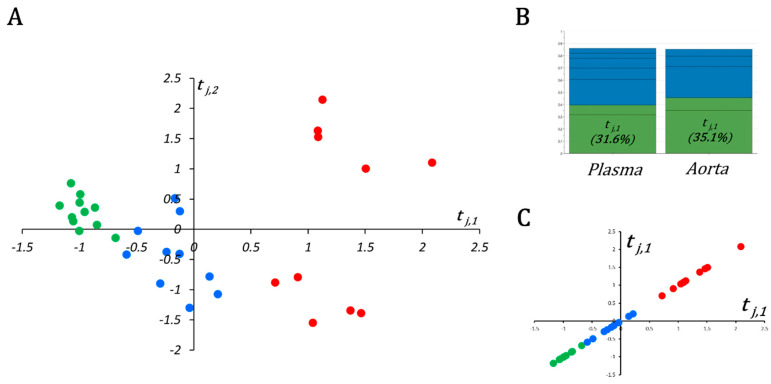
Scatter plot and metrics of the MOCA model. (**A**) Scatter plot of the first two globally joint components, t_j,1_ (x-axis) and t_j,2_ (y-axis); SHAM, HCD, and ANG groups are represented by green, blue, and red circles, respectively. (**B**) The first joint component (t_j,1_) explains more variance (>30%) than any other joint (i.e., t_j,2_) or unique component in plasma and aortic metabolomic blocks. (**C**) Separation between three compared groups becomes evident when samples are projected on t_j,1_ only instead of t_j,1_ and t_j,2_.

In this multiblock model, the OnPLS-based algorithm found two globally (or locally in the case of two blocks) joint components t_j,1_ and t_j,2_ (Figure 3A). The first joint component t_j,1_ explained up to 31.6% and 35.1% of the variance in the plasma and aorta metabolite matrices (i.e., X_pl_ and X_ao_), respectively, and was the most informative component among joint and unique components for both plasma and aortas (Figure 3B). Very interestingly, when samples were plotted on t_j,1_ (Figure 3C), they were perfectly ordered according to the atherogenic environment the mice were subjected to. Indeed, control mice had negative coordinates, while mice under both high-cholesterol diet and hypertensive insult, resulting in aortic aneurysms, had positive coordinates in t_j,1_. Mice fed with a high-cholesterol diet but without drug-induced hypertensive stress had near-zero coordinates in t_j,1_.

As far as groups were very strongly ordered in t_j,1_ following the “aneurysmal risk” (i.e., ANG > HCD > SHAM); we decided not to use a supervised algorithm such as OPLS-DA for plasma or aorta block. Instead, to appreciate variable importance in t_j,1_, we calculated the robust nonparametric Kendall τ correlation coefficient and associated *p*-values between metabolites and their loadings on t_j,1_. It should be noted that t_j,1_ is the same for both blocks (i.e., is a new attribute or variable with a unique realization for each mouse). The complete list of important metabolites (i.e., *p*-value equal or less than the α-threshold) for plasma and aorta blocs is given in Supplementary Data S3.

#### 2.4.2. Analysis of Plasma Block Metabolomic Signature of Aneurysmal Risk

**Figure 4:**
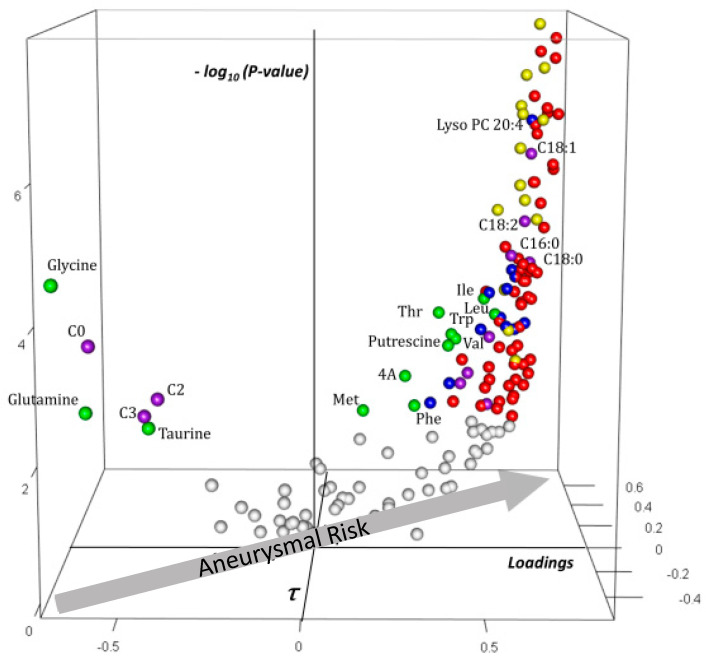
Three-dimensional volcano plot for plasma block according to t_1,j_. Coordinates for each metabolite represent loading (*x*-axis), Kendall τ coefficient (*y*-axis), and negative log-transformed *p*-values testing the null hypothesis τ =0 (*z*-axis). Only significant metabolites (i.e., *p* at most equal to the α-threshold calculated according to Benjamini–Hochberg procedure) have been colored. The arrow on the *x*,*y*-plane indicates that aneurysmal risk positively correlates with positive loadings and τ, as shown in the scatter plot of Figure 4. Each sphere represents one metabolite with the following color code: red: phosphatidylcholine species; yellow: sphingomyelin species; blue: lyso phosphatidylcholine species; violet: free and acylcarnitine species: green: amino acids and amino-acid-related metabolites. Carnitine is indicated by “C0”; acylcarnitines and lysophosphatidylcholine species are indicated by “C X:Y” and “Lyso PC X:Y” with X indicating the length of the acyl moiety and Y the number of double bonds in the acyl moiety, respectively. Abbreviations: Ile: isoleucine; Leu: leucine; Val: valine; Phe: phenylalanine; Met: methionine; Thr: threonine; 4A: α-aminoadipic acid; t4 OH-Proline: *trans*-4-hydroxyproline.

The plasma signature (Figure 4) showed increasing concentrations of phosphatidylcholine (PC), sphingomyelin (SM), lysophosphatidylcholine (LysoPC), and long-chain acylcarnitine (LCAC) species with aneurysmal risk. Along with lipidic molecules, seven amino acids—including the three branched-chain amino acids (BCAA); two aromatic amino acids, phenylalanine and tryptophan; threonine and methionine—and two amino-acid-derived metabolites (α-aminoadipic acid resulting from lysine catabolism and the polyamine putrescine, derived from ornithine) correlated positively and significantly with t_j,1_. In contrast, concentrations of six polar metabolites—three aminoacids (glycine, glutamine, and taurine), free carnitine (C0), and the shortest acylcarnitines C2 (acetylcarnitine) and C3 (propionylcarnitine)—decreased with aneurysmal risk.

**Figure 5:**
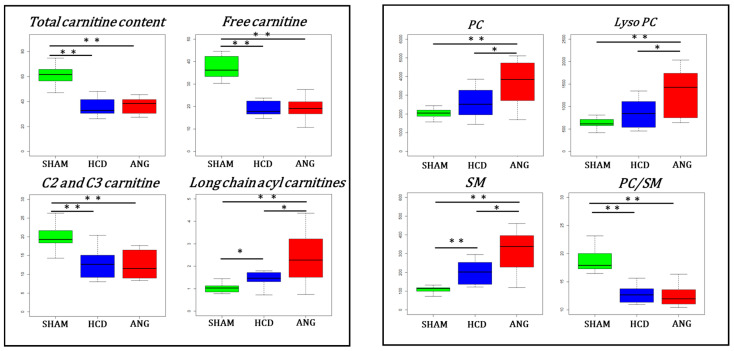
Box plots for metabolites and metabolite families involved in lipid metabolism and structure in the plasma block. Interquartile range has been colored green, blue and red for the SHAM, HCD, and ANG groups respectively. Mann–Whitney–Wilcoxon test was used for comparing group medians pairwise. Legend: total carnitine content: sum of free carnitine (C0) and all acylcarnitine species; long chain acylcarnitines: sum of all acylcarnitines with an acyl moiety of more than 12 carbons; C2 and C3 carnitine: sum of acetylcarnitine (C2) and propionylcarnitine (C3); PC: sum of all phosphatidylcholine species; Lyso PC: sum of all lysophosphatidylcholine species; SM: sum of all sphingomyelin species; PC/SM: ratio between all phosphatidylcholine species to all sphingomyelin species; *: *p* ≤ 0.05; **: *p* ≤ 0.01.

A closer look at some individual metabolites or metabolite families and ratios of biochemical interest in lipid metabolism is presented in Figure 5. In the plasma block, most significant changes related to carnitine and acylcarnitine concentrations appeared related to cholesterol diet with a significant lack of carnitine and decreased C2 and C3 acylcarnitines in both HCD and ANG groups. However, the median concentration of long-chain acylcarnitine species appeared to gradually increase with the aneurysmal risk. As for LCAC, median glycerophospholipids (PC, SM and LysoPC) concentration was progressively augmented according to the aneurysmal risk. Finally, the median of the PC/SM ratio was significantly decreased in the plasma of mice fed with a high-cholesterol diet. The envelope of VLDL and LDL is particularly enriched in SM, so this ratio inversely correlates to the concentration of atherogenic lipoproteins [24].

#### 2.4.3. Plasma Biomarker Research of Aortic Aneurysm

**Figure 6:**
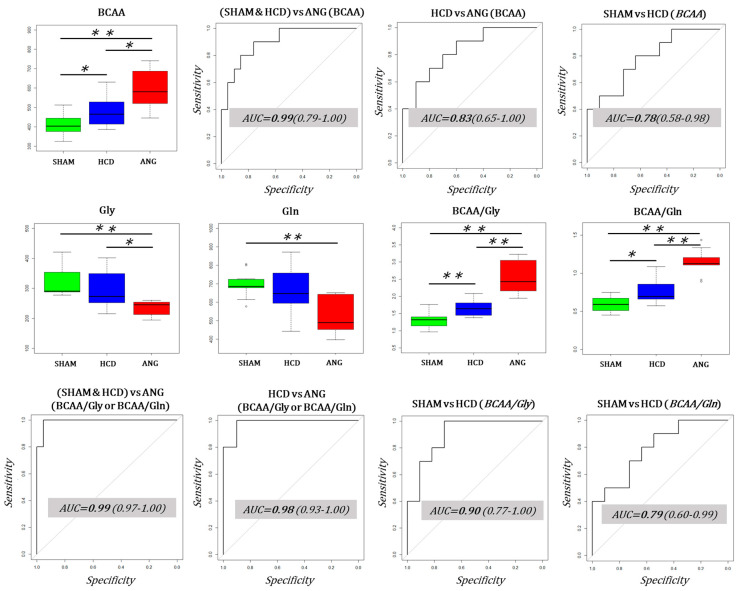
Performance of branched-chain amino acids (BCAA) and the ratios BCAA/glycine and BCAA/glutamine as classifiers for aortic aneurysm resulting from very high aneurysmal risk. Top panel: BCAA median concentration was significantly different between compared groups and increased with increasing aneurysmal risk. However, even when the median AUCs for the most important comparisons (ANG vs. no-ANG and HCD vs. ANG) were higher than 0.80, lower confidence intervals (LCI) included 0.8 in both comparisons. Middle panel: Median glycine (Gly) concentration was significantly decreased in t ANG group compared to the other two groups, while median glutamine (Gln) concentration was only significantly decreased in the SHAM group compared to the ANG group. Both BCAA/Gly and BCAA/Gln medians increased significantly with aneurysmal risk. Bottom panel: BCAA/Gly and BCAA/Gln performed very well as classifiers in discriminating no-ANG from ANG and HCD from ANG groups with AUC > 0.95 and LCI > 0.90. BCAA/Gly outperformed BCAA/Gln in discriminating SHAM from HCD groups. Interquartile range has been colored green, blue, and red for SHAM, HCD, and ANG groups, respectively. Mann–Whitney–Wilcoxon testing was used for comparing group medians pairwise. Legend: AUC: area under the ROC curve; *: *p* ≤ 0.05; **: *p* ≤ 0.01.

Using important plasma metabolites based on MOCA analysis, we intended to search for easily measurable biomarker candidates of very high aortic aneurysmal risk (Figure 6). We first evaluated the performance of BCAA as candidate biomarkers. Indeed, all three branched-chain amino acids—leucine, isoleucine, and valine—increased with aneurysmal risk, as depicted in Figure 4. Even when the median concentration of BCAA in SHAM, HCD, and ANG groups was significantly different, 95% confidence intervals included 0.80. Since glycine (Gly) and glutamine (Gln) concentrations decrease with aneurysmal risk, we calculated the ratio between BCAA and these two amino acids and determined their performance as biomarker candidates. We found that the ratio BCAA/Gly and the ratio BCAA/Gln had very good sensitivity and specificity in discriminating HCD and ANG groups (Figure 6). Both ratios performed identically in separating ANG from the other two groups. However, the BCAA/Gly ratio outperformed the BCAA/Gln ratio in discriminating HCD groups from SHAM groups.

#### 2.4.4. Analysis of the Aortic Block Metabolomic Signature of Aneurysmal Risk

**Figure 7:**
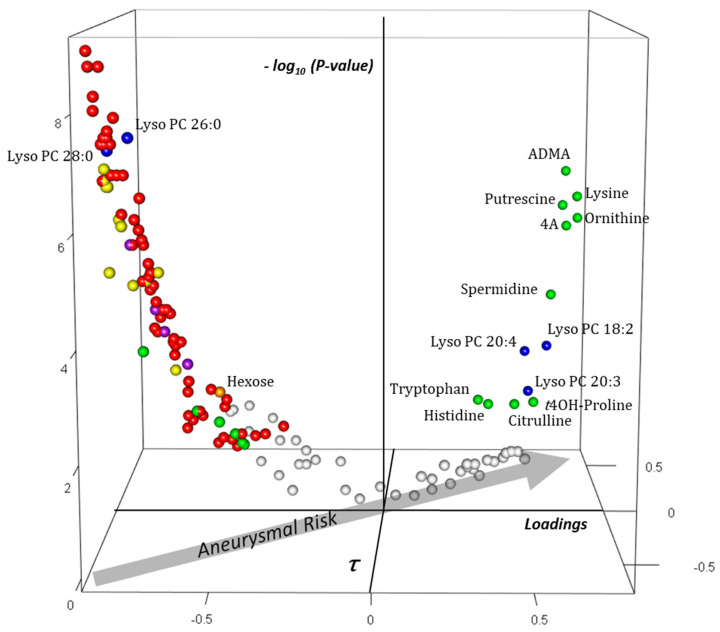
Three-dimensional volcano plot for the aortic blocks according to t_1,j_. Coordinates for each metabolite represent loading (*x*-axis), Kendall τ coefficient (*y*-axis), and negative log-transformed *p*-values testing the null hypothesis τ = 0 (*z*-axis). Only significant metabolites (i.e., *p* at most equal to the α-threshold calculated according to the Benjamini–Hochberg procedure) have been colored. The arrow in the *x*,*y*-plane indicates that aneurysmal risk positively correlates with positive loadings and τ, as shown in the scatter plot of Figure 4. Each sphere represents one metabolite with the following color code: red: phosphatidylcholine species; yellow: sphingomyelin species; blue: lyso phosphatidylcholine species; violet: free and acylcarnitine species; green: amino acids and amino-acid-related metabolites; orange: hexoses. Carnitine is indicated by “C0”; acylcarnitines and lysophosphatidylcholine species are indicated by “C X:Y” and “Lyso PC X:Y” with X indicating the length of the acyl moiety and Y the number of double bonds in the acyl moiety, respectively. Abbreviations: ADMA: asymmetric dimethylarginine; 4A: α-aminoadipic acid; *t*4OH-Proline: *trans*4-hydroxyproline.

In the aortic bloc (Figure 7), asymmetric dimethylarginine (ADMA), a potent inhibitor of nitric oxide synthase (NOS), was the metabolite which had the greatest positive *x*-, *y*-, and *z*-coordinates. It was followed by the polyamine putrescine and its precursor ornithine and by lysine and its catabolite α-aminoadipic acid. Concentrations of other amino acids (tryptophan, histidine, citrulline, and *trans*-4-hydroxyproline) were also raised by aneurysmal risk. Contrary to the plasma block, aneurysmal risk caused a decrease in most PC and SM species as well as lysophosphatidylcholine species with saturated acyl moieties in the aortic tissue. Among lipidic molecules, the concentration of only three lysophosphatidylcholine species with unsaturated acyl moieties was found raised in the aortic tissue with aneurysmal risk. Like in plasma, the concentration of some polar metabolites, including amino acids taurine and glycine and free and short-chain acylcarnitines (C0, C2, and C3), decreased with aneurysmal risk. The concentration of other amino acids (leucine, tyrosine, and methionine), carnosine, and the sum of hexoses was found also to decrease with the increase in aneurysmal risk.

**Figure 8:**
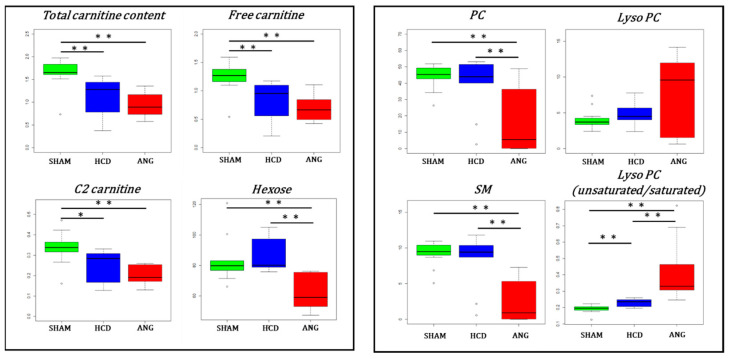
Box plots for metabolites and metabolite families involved in lipid metabolism and structure in the aortic block. Interquartile range has been colored green, blue, and red for the SHAM, HCD, and ANG groups respectively. Mann–Whitney–Wilcoxon testing was used for comparing group medians pairwise. Legend: total carnitine content: sum of free carnitine (C0) and all acylcarnitine species; C2: acetylcarnitine; hexose: sum of all L-hexoses, mainly D-glucose; PC: sum of all phosphatidylcholine species; Lyso PC (unsaurated/saturated): sum of all lysophosphatidylcholine species with an unsaturated acyl moiety divided by the sum of all lysophosphatidylcholine species with a saturated acyl moiety; SM: sum of all sphingomyelin species; *: *p* ≤ 0.05; **: *p* ≤ 0.01.

Paralleling plasma, free carnitine (C0) and acetylcarnitine (C2) were decreased in aortas of high-cholesterol diet fed mice (Figure 8) (to note that propionylcarnitine (C3) was not accurately measured in aortic tissue). There were no significant differences in LCAC in aortic tissue for the three levels of aneurysmal risk. However, median aortic hexose content (mainly D-glucose) was significantly decreased in ANG group compared to the other two groups (*p* < 0.001). As expected from the 3D volcano plot, PC and SM contents were significantly reduced in aortas from the ANG group. Interestingly, even when lysophosphatidylcholine content did not vary significantly among the compared groups, the dispersion of values around the median was very important in the ANG group compared to the SHAM and HCD groups, as evidenced by the large interquartile distance observed for this group. Furthermore, under no activation of phospholipase, a decrease in lysophosphatidylcholine concentration is expected in the ANG group following the drop in its precursor, phosphatidylcholine (PC). Phospholipase activity can be measured by the ratio of LysoPC to PC (LysoPC/PC). The median LysoPC/PC ratios calculated for the SHAM, HCD, and ANG groups were 0.079, 0.109, and 2.212, respectively (*p* = 0.024 for SHAM vs. HCD and *p* < 0.0001 for HCD vs. ANG).

Guided by the results from MOCA analysis showing increased concentration in aortic tissue of three lysophosphatidylcholines with unsaturated acyl moieties and decreased concentration of two lysophosphatidylcholines with saturated acyl moieties, with the increase in aneurysmal risk, we calculated the ratio of unsaturated-to-saturated lysophosphatidylcholines (Lyso PC U/S) as a marker of phospholipase A1 (PLA1) vs. phospholipase A2 (PLA2) activities. Indeed, when PLA1 acts on phosphatidylcholines, the resulting lysophosphatidylcholines very often has an unsaturated acyl moiety. The acyl moiety linked to the first carbon of phosphatidylcholines is very often saturated; the opposite occurs when PLA2 acts on phosphatidylcholines—the resulting lysophosphatidylcholines has in most cases a saturated acyl moiety. Medians for Lyso PC U/S ratio were 0.20, 0.24 and 0.34 for the SHAM, HCD, and ANG groups (*p* = 0.004 for SHAM vs. HCD and *p* < 0.0001 for HCD vs. ANG), respectively.

**Figure 9:**
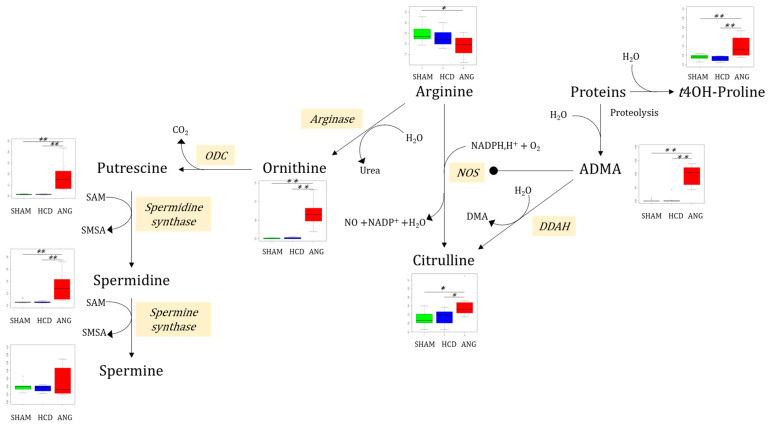
Main findings on polar metabolites in the aortic block and the arginine catabolic pathway. Interquartile range has been colored green, blue, and red for the SHAM, HCD, and ANG groups respectively. Mann–Whitney–Wilcoxon testing was used for comparing group medians pairwise. Legend: ADMA: asymmetric dimethylarginine; *t*4OH-Proline: *trans*4-hydroxyproline; NOS: nitric oxide synthase; ODC: ornithine decarboxylase; DDAH: dimethylarginine dimethylaminohydrolase; DMA: dimethylarginine; SAM: S-adenosyl methionine; SMSA: S-methyl-thioadenosine; *: *p* ≤ 0.05; **: *p* ≤ 0.01.

The analysis of the most important polar metabolites in the aortic bloc was carried out using arginine metabolic pathways (Figure 9). Indeed, even when median arginine concentration itself was significantly diminished only in the ANG group compared to the SHAM group, ornithine—the product of arginase reaction—was markedly increased in the ANG group compared to both the SHAM and HCD groups.

Polyamines putrescine and spermidine, resulting from ornithine decarboxylase (ODC) then spermidine synthase activities, were also considerably increased in the ANG group relative to the SHAM and HCD groups. Besides arginase, arginine could be oxidized by nitric oxide synthase (NOS), yielding nitric oxide (NO) and citrulline. Median citrulline concentration was significantly higher in the ANG group compared to the SHAM (*p* = 0.005) and HCD (*p* = 0.035) groups. As expected from MOCA analysis, median ADMA concentration was much higher in aortic tissue from ANG mice compared to the other two groups. Indeed, for this metabolite, all concentrations in the ANG group were at the upper bounds for the set of concentrations from non-ANG groups. ADMA results from proteolysis of protein with dimethylarginyl residues. Proteolysis of collagen-enriched extracellular matrix also results in *trans*-4-hydroxyproline (*t*4OH-Pro). Median concentration of this modified protein prolyl residue was significantly increased in the ANG group compared to the other two groups.

## 3. Discussion

We here present a deep metabolic phenotyping of a mouse model of aortic aneurysm generated by a high-cholesterol diet and angiotensin-II-induced hypertension. A profound metabolic remodeling was evidenced at both blood and aortic levels.

In blood, the metabolomic signature is marked by an overall increase concentration of phosphatidylcholines, lysophosphatidylcholines, sphingomyelins, and long-chain fatty acids, and by the increased concentrations of seven amino acids—including branched-chain amino acids—while free carnitine, short-chain acyl-carnitines, glycine, glutamine, and taurine are decreased. This hyperlipidemia is thus accompanied by a disruption of mitochondrial fatty acid oxidation (FAO) through the carnitine shuttle transport system as well as by an increased concentration of metabolic biomarkers of insulin resistance, such as branched-chain amino acids, long-chain acyl-carnitines, and α-aminoadipate. It should be noted that angiotensin II alone can promote insulin resistance [25]. Interestingly, the dyslipidemic profile is not only influenced by a high-cholesterol diet, but it is also further amplified by angiotensin II, as shown for long-chain acyl-carnitines, phosphatidylcholines, lysophosphatidylcholines, and sphingomyelins. The decrease in the ratio of phosphatidylcholine to sphingomyelins shows that this altered lipid profile may be directly related to the accumulation of VLDL and LDL since these atherogenic lipoproteins are particularly enriched in SM [24].

Also in plasma, BCAA/glycine and BCAA/glutamine ratios showed a high predictive power in terms of predictive sensitivity and specificity for aortic aneurysm compared to the other two SHAM groups. These ratios are therefore potential biomarkers of aortic aneurysm. Mechanistically, the increase in BCAA/Gln can be explained by an inhibition of muscular mitochondrial branched-chain aminotransferase (BCAT2) resulting in decreased BCAA catabolism and glutamine production. BCAT2 catalyzes deamination of three BCAA whilst transferring the amino group to α-ketoglutarate (α-KG), leading to the formation of branched-chain α-ketoacid and glutamate [26]. Glutamate can be eventually aminated to glutamine by glutamine synthetase. In this context, insulin resistance with enhanced—although incomplete—FAO increased acetyl-CoA production, which outpaces tricarboxylic acid (TCA) cycle flux (imbalance FAO/TCA cycle) with a relative lack of its intermediaries [27]. BCAT2 is inhibited by oxidative stress and high NADH/NAD^+^ ratio, both present in insulin resistance. The advantage of BCAT2 inhibition in muscle mitochondria would be sparing the α-KG for the TCA cycle, alleviating the imbalance between the FAO and TCA cycles. Furthermore, long-chain acylcarnitine species are toxic for many oxidative cells, including muscular cells, as evidenced by the intense rhabdomyolysis observed in patients with congenital disorders of long-chain FAO [28]. Glycine N-acyltransferase (GLYAT) catalyzes the formation of acyl-glycine species that are much less toxic than their respective acyl-carnitine derivatives [29]. The observed reduction in glycine concentration could be explained by an increased detoxifying activity of GLYAT in this context of incomplete FAO. Regarding the decrease in glutamine in blood, it is interesting to note that this amino acid is involved in macrophagic activation, energy homeostasis, oxidative stress, and angiogenesis and that it has been shown to be negatively correlated with atherosclerotic lesions [30]. It has also recently been shown that glutamine supplementation can reduce some atherosclerosis markers in athletes [31]. Taurine and glycine, which are also lowered in the blood in our study, are also known to be protective against atherosclerosis, while the increase in branched-chain amino acids is deleterious for vascular pathophysiology [32]. The decrease in carnitine in the blood of our model is also a vascular damaging factor, carnitine supplementation being protective against atherosclerotic lesions in hypercholesterolemic rabbits [33].

In aortas, the evolution of lipid profile under an increasing aneurysmal risk was strikingly opposed to that seen in plasma with a decrease in many phosphatidylcholine and sphingomyelin species concentration. These differences could be attributed to the decrease in vascular cellularity and to an increase in lipid oxidation in the aortic wall due to aneurysmal remodeling. Although only nonoxidized lipids can be measured by our metabolomic approach, the hypothesis that mainly lipids with oxidized acyl moieties are present in the aneurysmatic aortic wall is very likely. Also, tissular phospholipids were found essentially in cell membranes and organelles but the extracellular matrix is a polar matrix composed almost of polar metabolites and proteins. It is well-documented that cellularity in abdominal aortic aneurysms is deeply decreased [34].

This vascular remodeling is also evidenced, specifically in the aneurysm group, by the increased concentration of hydroxyproline generated by collagen degradation. The unsaturated-to-saturated lysophosphatidylcholine ratio is highly discriminating in the aneurysm group compared to the other two groups, suggesting an involvement of phospholipase A2 activity, as it has already been shown in aortic aneurysms [35]. Also, in damaged endothelium platelet-derived phospholipase A1 (PLA1) activation will release lysophosphatidylcholine and then phosphatidic acid (PA) a potent enhancer of mineralization and osteogenic transformation in the aortic wall [36]. Indeed, PA has been recently postulated as an early marker of aortic dissection [37]. The carnitine profile is also reversed compared with blood, showing a decrease in free (C0) and acetyl-carnitine (C2) without an increase in long-chain acyl-carnitines. Relatively decreased hexose concentration in the group with aortic aneurysms probably reflects enhanced glycolysis in the aneurysmal aorta.

The aortic profile is also marked by an alteration of the nitric oxide metabolic pathway, with increased consumption of arginine leading to increased citrulline production by NO synthase and of ornithine by arginase, specifically in the group developing aneurysms. Arginase may have detrimental effects on fibroproliferative vascular diseases through its ability to enhance vascular smooth muscle and endothelial cell proliferation by promoting polyamine biosynthesis [38]. In addition, arginase interferes with NO metabolism by competing with NO synthase for their common substrate, arginine. The large increase in concentration in aneurysms of ADMA, also provided by protein degradation, is probably a major pejorative factor identified here because this biomarker is associated with an increased risk of vascular dysfunction. ADMA is a strong inhibitor of NO synthase and a critical regulator of NO metabolism in vascular homeostasis [39]. The production of the three polyamines, which have pleiotropic roles in cellular growth and proliferation is also strongly activated in aneurysms. Interestingly, increased polyamine concentrations have already been reported in the aortas of patients affected by bicuspid aortic valve, a congenital malformation that is associated with an increased risk of aneurysm [40]. These authors reported that polyamines may have both beneficial and detrimental effects on aortic pathophysiology by interfering with NO metabolism, protein stabilization, and antioxidant and anti-inflammatory activities. Thus, our study shows that NO metabolism is strongly disturbed in aortic aneurysms; NO being known to be involved in the progression of abdominal aortic aneurysms at least in part through the metalloproteinases whose pathophysiological importance in this pathology is well known [41].

Lysine, an essential amino acid, is also greatly increased in aortic aneurysm signature. Interestingly, such accumulation of lysine has already been shown in pressure-overload-induced cardiac hypertrophy in rats [42], and an involvement of this amino acid in vascular calcification, which would be attractive in aortic aneurysms, has been reported [43].

However, we can identify some limits to our study. Indeed, the group of Ldlr^−/−^ mice receiving angiotensin II without a hypercholesterolemic diet is missing, and this could induce a bias in relation to the analysis of the results by omitting the role of hypertension per se. Nevertheless, as previously demonstrated by Cassis et al., angiotensin II promotes the development of abdominal aortic aneurysms (AAAs) independently of the increase in blood pressure in hypercholesterolemic mice such as Ldlr^−/−^. Indeed, high blood pressure is not the direct cause of the increases in atherosclerotic lesion size that occur during chronic subcutaneous infusion of angiotensin II [44]. Several other studies have inferred dissociation of blood pressure and ANG-II-induced AAAs, such as the administration of doxycycline in Ldlr^−/−^ mice (matrix metalloproteinases inhibitor), reduced the AAAs mediated by angiotensin II induction without having an effect on blood pressure [45]. Moreover, the addition of a lipid rich diet increases AAA incidence in Ldlr^−/−^ mice [46], suggesting an effect of hypercholesterolemia on the development of the pathology, other than the isolated induction of angiotensin II. After considering all these aspects, we chose to compare the AAA group with the SHAM group fed with the standard diet, allowing us to limit the number of control groups.

To conclude, in aortas, experimental aneurysms are accompanied by increased collagen remodeling, disruption of lipid metabolism with increased phospholipase A1 and A2 activity, increased glucose, NO and lysine metabolisms, and increased polyamine production. Given its highly significant contribution to the model, ADMA produced by vascular remodeling is a detrimental factor that may interfere with NO production. In blood, the major hyperlipidemia observed is probably more related to the experimental diet and its consequences at the organism level, but with decrease concentrations of several metabolites known to play a protective role against atherosclerosis such as glutamine, glycine, taurine and carnitine, and increased concentrations of the deleterious branched amino acids, the BCAA/glycine and BCAA/glutamine ratios show good predictive power of the aneurysm. Decreased glycine, taurine, and free carnitine (C0) in the aortic wall with the increase in aneurysmal risk could stem from a systemic lack of these metabolites, as reflected by their lower plasmatic concentrations in the angiotensin-II-treated group. Furthermore, in mice under hypertensive insult, free carnitine deficiency could impair FAO in all tissues, as evidenced by diminished plasmatic and aortic acetyl-carnitine (C2) concentrations. Additionally, diminished concentration of hexoses in damaged compared to nonaneurysmatic aortas probably indicates a shift from FAO to glycolysis induced by carnitine deficiency in this tissue. It would be very interesting in future studies to compare the time to event (aneurysm rupture or dissection) in angiotensin-II-treated mice fed with standard or with glycine-, taurine-, and carnitine-enriched diet.

## 4. Materials and Methods

### 4.1. Animal Models and Diet

Male Ldlrtm1Her (Ldlr^−/−^) mice on a C57BL6/J background aged 4 to 6 months were used [47]. Mice were divided into two groups, each including 10 control mice (SHAM), were fed with a standard diet (U8959P 0022 Safe-Diet) or a high-cholesterol diet (+1.25% cholesterol, U8959P 0022 Safe-Diet) for 28 days. The third group is the aneurysm group, with 10 hypertensive mice fed with a high-cholesterol diet for 28 days. Mice were housed under barrier conditions with food and water provided ad libitum.

For the metabolomic experience 31 mice were included. The SHAM group included 11 mice and the other two groups (high-cholesterol diet fed mice or HCD group and high-cholesterol fed plus angiotensin-treated mice or ANG group) included 10 mice each.

### 4.2. Hypertension Induction

Hypertension was induced by subcutaneous implantation of osmotic mini-pumps (ALZET^®^ 2004, with angiotensin II (Angiotensin II acetate salt #H-1705.0100 Bachem^®^, Torrance, CA, USA) dissolved in NaCl 0.09%) in the dorsal region under isoflurane anesthesia to achieve a delivery rate of 1000 ng·kg^−1^ of body weight·min^−1^ for 28 days, as previously described in Ldlr^−/−^ mice [14,48,49].

### 4.3. Blood Pressure and Bodyweight Measurement

Systolic blood pressure was measured in conscious mice using a computerized tail cuff method (BP-2000; Visitech Systems, Apex, NC, USA). All mice were acclimated to the system for 1 week prior to the start of the study. After habituation, measurements were taken at baseline (before surgery, D0), during treatment (D14), and before animal sacrifice (D28). Each day, systolic blood pressure average was taken over 15 successive measurements, of which an average over three days was taken for each measurement. Measurement of mouse bodyweight was taken each week.

### 4.4. Sacrifice, Samples Extraction and Lipid Profil

After 28 days of treatment, animals were sacrificed by CO_2_ inhalation after 3 h of fasting. The blood was collected by cardiac puncture and packaged in a heparinized tube, centrifuged (4 °C, 1699× *g*) to collect 400 μL of plasma, and frozen at −80 °C until assayed. 

The heart was weighed and related to mouse weight to assess cardiac hypertrophy. Aortas were dissected from perivascular fat in saline PSS (130 mM NaCl, 78.8 mM KCl, 1.2 mM MgSO_4_(7H_2_0), 14.9 mM NaHCO_3_, 5 mM HEPES, 1.2 mM KH_2_PO_4_, D-glucose 11 mM) at 4 °C and at a physiological pH of 7.4. Samples were then packaged in a dry tube at −80 °C.

### 4.5. Lipid Profile

Lipid profile was made on heparinized plasma and included the dosages of total cholesterol (CT), HDL-cholesterol (HDL-c) and triglycerides (TG). LDL-cholesterol (LDL-c) concentration was calculated by the Friedewald formula: LCL-c = CT-HDLc-(TG/2.2) (mmol/L).

### 4.6. Histomorphologic Analysis

After dissection, abdominal aortas were placed in Tissue-tek^®^ or O.C.T (Optimal Cutting Temperature Compound, Thermo Scientific, Waltham, MA, USA) storage solution, frozen in nitrogen vapors, and stored at −80 °C. The blocks were cut transversely with 7 μm thickness using a Cryostat^®^ (Leica CM3050 S) at −25 °C. The sections were then placed on polarized ThermoFrost slides (SuperFrost Ultra Plus, Thermo Scientific) and stored at −80 °C until use.

Abdominal aorta sections were stained with orcein solution and counterstained by hematoxylin and eosin. Lipid deposition in the aortic wall was measured by Oil Red O staining. Image acquisitions were carried out by the microscopic analysis service (SCIAM, Angers University, Angers, France) of the Angers University Hospital using the Keyence microscope (VHX-7000) and the software integrated into the microscope. Total area, lumen, media, adventitia, and Oil-Red-O-positive areas of each artery were measured in mm^2^ using Image J software.

### 4.7. Sample Preparation for Metabolomic Analysis

Frozen aortas were weighed on a precision scale and transferred to a 2.0 mL homogenization Precellys tube prefilled with 1.4 mm diameter ceramic beads and 60 μL of cold methanol. Samples were kept on dry ice during the weighting to avoid thawing. Tissues were homogenized with two grinding cycles of 6600 rpm for 20 s spaced by 20 s followed by a third grinding cycle of 6000 rpm for 30 s using a Precellys homogenizer (Bertin Technologies, Montigny-le-Bretonneux, France) kept in a room at +4 °C. The supernatant was recovered after centrifugation of the homogenate at 16,000× *g* and kept at −80 °C until mass spectrometry analysis. Plasma samples were kept at −80 °C until mass spectrometry analysis.

### 4.8. Mass Spectrometry Analysis of Plasma and Aortas Extracts 

We applied a targeted, quantitative metabolomic approach to plasma and tissue extracts by using the Biocrates AbsoluteIDQ p180 Kit (Biocrates Life Sciences AG, Innsbruck, Austria). This kit, in combination with an AB Sciex QTRAP 5500 (SCIEX, Villebon sur Yvette, France) mass spectrometer, enables quantification of up to 188 different endogenous molecules, including 40 acylcarnitines, 21 amino acids, 21 biogenic amines, 90 glycerophospholipids, 15 sphingolipids, and the sum of hexoses (mainly D-glucose in mammalian tissues). More information about this technology can be found at https://biocrates.com/absoluteidq-p180-kit/. (the content of this webpage concerning p180 technology was verified on 19 April 2022) Flow injection analysis with tandem mass spectrometry (FIA-MS/MS) was used for quantifying acylcarnitines, glycerophospholipids, sphingolipids, and sugars, whereas liquid chromatography (LC) allowed the separation of amino acids and biogenic amines before detection with tandem mass spectrometry (LC-MS/MS). Samples were prepared according to the Biocrates Kit User Manual. In brief, after thawing on ice, 10 μL of each sample (aorta homogenate supernatant or plasma) was added to the center of the filter placed on the upper wall of the well in a 96-well plate. Metabolites were extracted in a methanol solution by using ammonium acetate after drying the filter spot under nitrogen flow and derivatizing with phenylisothiocyanate for the quantification of amino acids and biogenic amines. Extracts were finally diluted with MS running the solvent before FIA and LC-MS/MS analyses. After validation of the three levels of quality control used with the kit, the metabolite concentrations inferred were used for statistical analyses. For aortas, each metabolite concentration obtained with the kit (raw concentration) for a given mouse was divided by the weight of the aorta used in the analysis, and the volume of methanol (60 μL) was kept constant for sample grinding (i.e., metabolite extraction).

### 4.9. Statistical Analysis of Metabolomic Data

#### 4.9.1. Univariate Analysis

Prior to statistical analyses, only metabolites with more than 70% of their concentration values in the dynamic range were taken into consideration. Data were then stored in two matrices named X_pl_ and X_ao_ for plasma and aorta matrices, respectively. These two matrices have dimensions m × k and m × l, where *m* is the number of samples (i.e., mice) and k and l represent the number of metabolites correctly measured in plasma and aortas, respectively. Nonparametric univariate analysis (Mann–Whitney–Wilcoxon test) was carried out to detect significant variation in median metabolite concentrations and metabolite ratios and sums in both matrices between groups of mice pairwise. Benjamini–Hochberg correction was used for correcting risk I inflation due to multiple comparisons and to maintain false discovery rate (FDR) less or equal than 5%. Wilcoxon testing was carried out using the function *wilcox.test* in R. Kendall’s τ for nonparametric correlation and its associated *p*-values were calculated using function *cor.test* in R software.

We determined the performance of discriminant classifiers (metabolites or metabolite sums or ratios) by calculating the area under the ROC curve (AUC) associated with the logistic regression model including these classifiers. Logistic regression and calculation of AUC and its 95% confidence interval using nonparametric Delong’s method were carried using the package *pROC* in R software [50]. A cut-off of 0.80 was fixed for deciding whereas the classifier had good performance (AUC ≥ 0.8) or not (AUC < 0.8). R software is available from https://www.R-project.org/ (functionality verified on 19 April 2022).

#### 4.9.2. Multivariate Analysis

Plasma and aorta metabolomic data were simultaneously analyzed by the multiblock orthogonal component analysis (MOCA) method [51]. MOCA is an unsupervised multivariate embodiment of the OnPLS algorithm, where OnPLS stands for orthogonal partial least squares, for *n* data matrices (in our case *n* = 2: plasma and aorta data matrices). MOCA splits information structures residing in data into correlated (joint) and uncorrelated (unique) sources of variabilities. Globally joint (or locally joint in the case of only two blocks) information is systematic structure (in the sense of principal component analysis, i.e., variance explanation) found in both plasma and aorta data blocks, whilst unique information is additional systematic structure found only in one (plasma or aorta) single data block. Roughly speaking, when a locally joint latent variable (t_j_) is projected in the column space of X_pl_ and X_ao_, then a significant amount of variance in each block is captured by t_j_. MOCA is implemented in SIMCA software, version 16 (available from www.sartorius.com/umetrics, 19 April 2022) using default procedures for data pretreatment, i.e., all variables were mean-centered and scaled to unit variance prior to model calculations.

## Figures and Tables

**Table 1:** Lipid profile in *Ldlr*^−/−^ mice with or without hypercholesterolemic diet and angiotensin II treatment.

	Normal Diet (*n* = 7)	High-Cholesterol Diet (*n* = 7)	High-Cholesterol Diet + Angiotensin II Treatment (*n* = 2–5)
Plasma cholesterol (mmol/L)	13.96 ± 1.38	11.09 ± 2.03	23.4 ± 1.4
Plasma triglycerids (mmol/L)	4.58 ± 0.83	0.99 ± 0.1 *	2.27 ± 1.07
Plasma HDLc (mmol/L)	1.06 ± 0.18	0.39 ± 0.08	0.94 ± 0.37
Plasma LDLc (mmol/L)	6.82 ± 1.29	10.24 ± 1.97	23.4 ± 1.041 *
LDLc/HDLc Ratio	9.1 ± 3.16	29.84 ± 5.18	80.69 ± 32.76
Total cholesterol/HDLc	15.72 ± 2.679	32.43 ± 5.425	117 ± 7

Legend: Data are given as the mean ± SEM. Kruskal–Wallis tests with Dunn correction were used (* *p* < 0.05 compared to *Ldlr*^−/−^ mice fed with a normal diet). HDLc: circulating high-density lipoprotein; LDLc: circulating low-density lipoprotein.

## Data Availability

Not applicable.

## Supplementary Materials

Supplementary materials can be found at https://www.mdpi.com/article/10.3390/ijms23126387/s1.

## Abbreviations

4A	α-aminoadipic acid
AAA	Abdominal aortic aneurysm
ADMA	Asymmetric dimethylarginine
ANG	Angiotensin II
BCAA	Branched-chain aminoacids
BCAT2	Branched-chain amino acid transaminase 2
C0	Free carnitine
C2	Acetylcarnitine
C3	Propionylcarnitine
DDAH	Dimethylarginine dimethylaminohydrolase
DMA	Dimethylarginine
FDR	False discovery rate
Gln	Glutamine
Gly	Glycine
GLYAT	Glycine N-acyltransferase
HCD	High-cholesterol diet
HDLc	Circulating high-density lipoprotein
Ile	Isoleucine
LC	Liquid chromatography
LCAC	Long-chain acylcarnitine
LDLc	Circulating low-density lipoprotein
LDLr	Low-density lipoprotein receptor
Leu	Leucine
LysoPC	Lysophosphatidylcholine
Met	Methionine
MMP	Matrix metalloproteinase
MOCA	Multiblock orthogonal component analysis
NAD	Nicotinamide adenine dinucleotide
ND	Normal diet
NOS	Nitric oxide synthase
ODC	Ornithine decarboxylase
PC	Phosphatidylcholine
Phe	Phenylalanine
PLA1	Phospholipase A1
SAM	S-adenosyl methionine
SBP	Systolic blood pressure
SM	Sphingomyelin
SMSA	S-methyl-thioadenosine
t4 OH-Proline	*Trans*-4-hydroxyproline
TCA	Tricarboxylic acid
Thr	Threonine
TIMP	Tissue inhibitor of metalloproteinase
Val	Valine
VLDL	Very-low-density lipoprotein
VSMC	Vascular smooth muscle cell
α-KG	α-ketoglutarate
